# C-Band 30 W High PAE Power Amplifier MMIC with Second Harmonic Suppression for Radar Network Application

**DOI:** 10.3390/mi13122079

**Published:** 2022-11-26

**Authors:** Fan Yang, Leijun Song, Yuehang Xu

**Affiliations:** 1 School of Electronic Science and Engineering, University of Electronic Science and Technology of China, Chengdu 611731, China; 2 Microwave Technology Research and Development Center, Beijing Institute of Radio Measurement, Beijing 100854, China

**Keywords:** radar network, power amplifier (PA), microwave monolithic integrated circuit (MMIC), additional power efficiency (PAE), second harmonic suppression (SHS)

## Abstract

In order to meet the application requirements of radar networks for high efficiency and high second harmonic suppression (SHS) of power amplifiers, this paper proposes a C-band 30 W power amplifier (PA) microwave monolithic integrated circuit (MMIC) based on 0.25 μm gallium nitride (GaN) high electron mobility transistor (HEMT) process. The proposed PA uses a two-stage amplifier structure to achieve high power gain. A topology with SHS is designed in the output-matching network. Besides, the large signal model load pull simulation and the harmonic control technology in the output stage are used to improve efficiency. The high-power additional efficiency (PAE) and high SHS of the PA MMIC are achieved simultaneously. In the 5–6 GHz frequency range, multiple indicator measurements of the proposed PA show that output power is over 45 dBm, the PAE is more than 57%, the SHS exceeds 45 dBc, the power gain is greater than 24 dB, which are conducted under the condition of 100 μs pulse width and 10% duty cycle. In addition, the size of the PA MMIC, including bonding pads, is 3.3 × 3.1 mm^2^.

## 1. Introduction

The radar network technology increases the degree of freedom of the system through reasonable configuration and optimal deployment of multiple radars and greatly improves the ability of signal interception and target detection in the coverage area [1]. The radar network system comprises multiple decentralized transmitting and receiving stations, which has obvious advantages in anti-stealth, anti-jamming, target positioning, and tracking [2]. Each radar in the radar network system can not only work independently but also work together with other radars to form a unified whole, which enhances the flexibility of the system. The mutual interference between radars in the network is required to be as small as possible, so a clear requirement is put forward for the harmonic energy generated by components.

With the improvement of GaN semiconductor technology, the research on GaN power amplifiers (PA) has made great progress in recent years. It has gradually replaced LDMOS PA in the application field. The GaN PA is widely used in detection radar, satellite communication, electronic jammer system, solid-state transmitter, and other industry fields [3,4,5,6,7,8,9,10,11]. Compared with other semiconductor processes, such as CMOS, SiGe, GaAs, InP, etc., GaN devices have higher junction temperature, higher breakdown voltage, and current density. Therefore, under the same size conditions, GaN power devices generate more output power. In addition, GaN on SiC devices can show good thermal properties mainly due to the high thermal conductivity of SiC. In fact, the PA designed with GaN high electron mobility transistor (HEMT) technology not only has the characteristics of high output power, broadband, and high efficiency but also has the characteristics of high voltage and low current of GaN power devices. These advantages simplify the secondary power conversion unit, thereby alleviating a series of problems caused by large current transmission.

In the radar transceiver module, PA is one of the most important circuits. Its performance indicators, such as output power, gain, efficiency, etc., will directly affect the power consumption of the transmitter, and its cost proportion is also the largest in the transceiver system. The characteristics of PA will directly affect the power consumption, heat dissipation design, and weight of the radar system [12]. In many previous research reports, high power and high efficiency have always been the research focus of Pas [13,14,15,16,17,18,19,20,21,22]. In the classic saturated Class A, Class B, Class AB, and Class C PAs [23,24,25], it is found by comparison that Class AB PAs can simultaneously take into account the main technical indicators such as bandwidth, output power, efficiency and linearity, and are widely used in radar engineering products [26,27,28].

The harmonic control PAs includes class E, class F, inverse class F, and class J [29,30,31,32,33,34,35], which are mainly based on the theory of waveform engineering. Its main feature is that it can greatly reduce the energy consumption of power devices themselves so as to obtain higher output efficiency. It is a type of high-efficiency PA that is widely studied. However, due to its poor bandwidth and linearity and other technical indicators, it is relatively less used in a high-power radar system.

When the input signal of the PA keeps increasing, the PA will produce many harmonic components because of entering the nonlinear region. Although harmonics can be eliminated with additional filters, high-power filters are large in size and introduce additional insertion loss, which affects the volume, output power, and efficiency of the transmission channel. Therefore, it is necessary to consider harmonic suppression from the point of PA design without using filters. 

The key point of PA MMIC design is the design of the matching network. The matching network mainly affects the port return loss, bandwidth, efficiency, and current consumption of the PA [36]. Output matching network mainly focuses on low loss, high efficiency, and output power flatness. The gain flatness and power drive ratio of the amplifier are greatly affected by the intermediate-stage matching network. The input matching network has a great influence on the overall stability and input voltage standing wave ratio of the PA. Compared with the input signal, the output signal has more harmonic components, which is an interference signal. Due to the frequency selection effect of the matching network, a proper design of the output matching network can reduce the harmonic level of the PA in the nonlinear working state and help to improve the efficiency.

However, most of the previous academic research has focused on high power and high PAE [10,22]. There has been little research on harmonic suppression, especially in the brand-new C-band PA MMIC [20,36]. Among the most advanced C-band GaN MMIC products, only [26,28] focus on the SHS performance. The products [26] and [28] realize the SHS with 34 dBc and 29 dBc, respectively. In order to meet the needs of the radar network, the goal of this paper is to achieve a 30 W monolithic PA MMIC in the 5–6 GHz frequency range, with PAE greater than 55% and SHS greater than 40 dBc.

This paper designs and implements a C-band 30 W PA MMIC with a PAE greater than 57%, which is used in the C-band transceiver module. Through harmonic load pull simulation, the optimal output load impedances of transistors at the fundamental frequency and second harmonic frequency are obtained, respectively [37,38,39]. By optimizing the frequency selection characteristics of the output matching network, a better SHS performance is achieved. The measured results show that the PAE of the PA MMIC is 57.2–62.6%, the saturated output power is 45.3–45.9 dBm, the power gain is more than 24 dB, and the SHS is 45–48 dBc in the 5–6 GHz frequency bandwidth. These indicators of the proposed C-band PA are suitable for the radar network system application.

## 2. Design Methods

The C-band 30 W high-efficiency HPA MMIC with high SHS was designed based on 0.25 µm GaN HEMT technology. The transistors of the GaN HEMT have excellent breakdown voltage characteristics of more than 120 V, a cutoff frequency (*f*_T_) of about 24 GHz, and a saturation output power density of 5.6 W/mm under the drain voltage bias of 28 V at 5.5 GHz. The interconnection line has two layers of metal. The current withstand capacity of the first layer of metal is 6 A/mm, and the current withstand capacity of the second layer of metal is 24 A/mm. The air bridge connection mode was used at the intersection of two metals. The design aims to achieve a high PAE GaN PA MMIC, which has an output power of 45 dBm (30 W), a power gain of more than 22 dB, a high SHS of 45 dBc, and a high PAE of over 55% in the 5–6 GHz frequency range.

**Figure 1:**
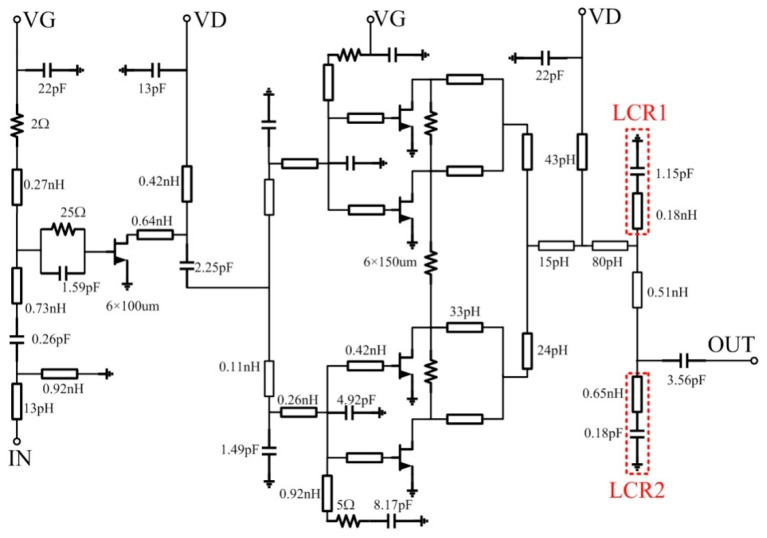
Schematic of the proposed C-band 30 W GaN PA MMIC with half-side circuit.

Half of the schematic topology of the proposed PA MMIC with two stages is described in Figure 1. The total output stage gate width is determined according to the power density of the HEMT and the required saturation output power of the PA. The number of stages of the PA is determined by the required power gain. The design of the driver stage also affects the PAE of the entire PA. The gate width of the drive stage transistor must be selected according to the input power required by the output stage transistor. The drive stage transistor needs to provide enough drive power for the output stage transistor, and the drive stage transistor cannot be deeply compressed. In order to improve the stability of the circuit, a small resistance connected in series between adjacent cells of the output stage transistor can effectively suppress odd mode oscillation, improve signal crosstalk, and help improve the synthesis efficiency. The drive stage transistor gate bias circuit uses an RC network to enhance the overall stability of the proposed PA. As a part of the matching circuit, the drain bias line needs to consider whether the line width can withstand the corresponding current. Because of the large current value, the inductance in the drain feed matching is realized by double metal transmission lines. The compact second harmonic suppression resonators LCR1 and LCR2 are added to the output stage matching network.

**Figure 2:**
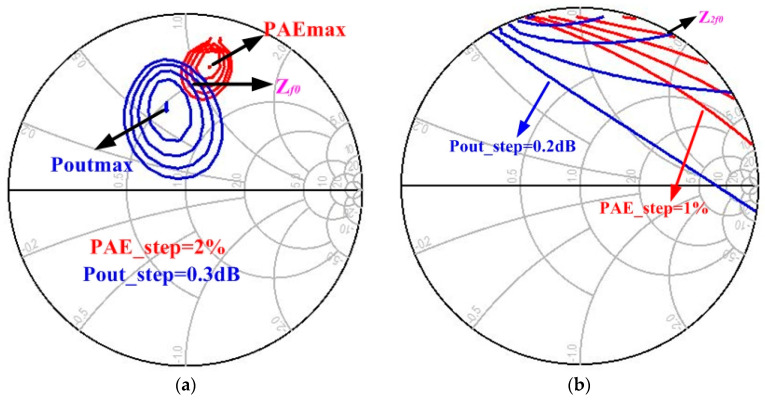
The fundamental and second harmonic impedance load pull simulation of the 6 × 150 µm transistor: (**a**) Fundamental impedance load pull simulation; (**b**) Second harmonic impedance load pull simulation.

To achieve high efficiency, fundamental and second harmonic impedance load pull simulations of the 6 × 150 µm transistor were performed, with the goal of obtaining optimal impedance at a fundamental and second harmonic frequency, as shown in Figure 2. All the eight 6 × 150 µm output stage transistors were operated in class AB bias condition, with a drain voltage of 28 V and a gate voltage of −2.2 V. The static current I_ds_ of the PA MMIC is 1.05 A, which can be completely matched with Equation (1) [36]. The variable parameters of HMET have the following meanings: W is the gate width, µ is the electron mobility, ε is the dielectric constant, L is the channel length, and d is the barrier thickness.

I_ds_ = Wµε(V_gs_ − V_th_)^2^/2Ld (1)

The load pull simulation steps adopted are as follows:

Step 1: the source impedance of the transistor is fixed at 10 ohms, and then the fundamental load traction simulation is carried out. After the output power and efficiency are compromised, the best load impedance value *Z*_opt1_ is selected as the fundamental load impedance. 

Step 2: The load impedance of the transistor is fixed at the optimized load impedance *Z*_opt1_ found in step 1, and then the source pull impedance simulation is carried out, and the best value Z_S1_ of the source impedance is determined.

Step 3: The impedance of the input terminal is fixed at the optimized source pull impedance *Z*_S1_ found in step 2, and then the load pull simulation is carried out to find out the best value of the load impedance *Z*_opt2_. 

Step 4: Step 2 and 3 are repeated until the source impedance and load impedance converge to the fixed impedance values.

The following impedance values are obtained from the above method. The source pull input impedance is taken as *Z*_S_ = 6.8 + j*5.2 Ω, the load pull fundamental impedance is taken as *Z*_f0_ = 23.6 + j*46.7 Ω, and the load pull second harmonic impedance is taken as *Z*_2f0_ = 1.2 + j*65.6 Ω. After the second harmonic load pull simulation, the maximum PAE is increased by 6% compared with only the fundamental load pull simulation. Finally, the output power of a single output stage transistor is 37.0 dBm, and the PAE is 77%.

**Figure 3:**
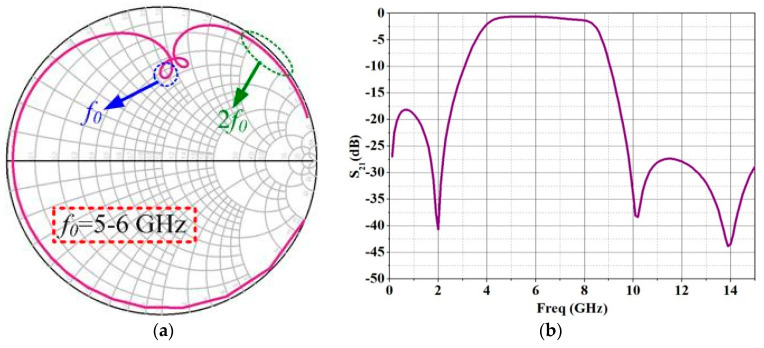
Simulation results of the proposed output matching network: (**a**) Input impedance of the proposed output matching network; (**b**) *S*_21_ of the proposed output matching network.

After the load pull simulation was completed, the output matching network was optimized according to the optimal load impedance. Figure 3 shows the impedance matching characteristics of the designed output matching network. Figure 3a shows that the designed output matching network is very close to the optimal fundamental impedance and the optimal second harmonic impedance in the C-band operating frequency range of 5–6 GHz. Figure 3b shows the low insertion loss of 0.6 dB in the 5–6 GHz frequency range and the second harmonic suppression characteristics in the 10–12 GHz range. Therefore, the design of the output matching network has the characteristics of low insertion loss and second harmonic suppression on the basis of achieving the optimal impedance. Inductance-capacitance series resonance introduces two transmission zeros within the second harmonic frequency range of the output matching network, and the resonant frequencies are in Equation (2).

*F*_z_ = (*LC*)^−1/2^/2π (2)

The SHS resonators, LCR1 and LCR2, are resonating at the frequencies of 10 GHz and 14 GHz, respectively.

**Figure 4:**
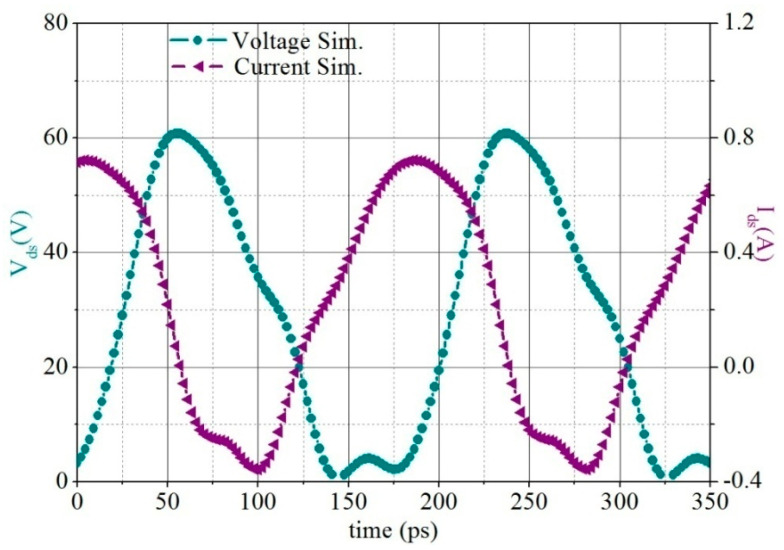
Time domain waveform simulation of the output stage GaN HEMT’s drain voltage and current.

Figure 4 shows the time domain waveform simulation curve of the drain voltage and current of the output stage transistor. It can be seen that the voltage and current have a small overlap, which is conducive to improving the PAE of the PA MMIC.

The drive stage was designed in the same way as the above load pull simulation. The drive stage adopted two 6 × 100 um transistors. Each transistor provides more than 34.3 dBm of drive power and more than 15 dB of power gain. The gate width ratio of drive stage and output stage was 1:6. The drive stage ensures sufficient output power to drive the output stage while maintaining high efficiency.

## 3. Measurement Results

**Figure 5:**
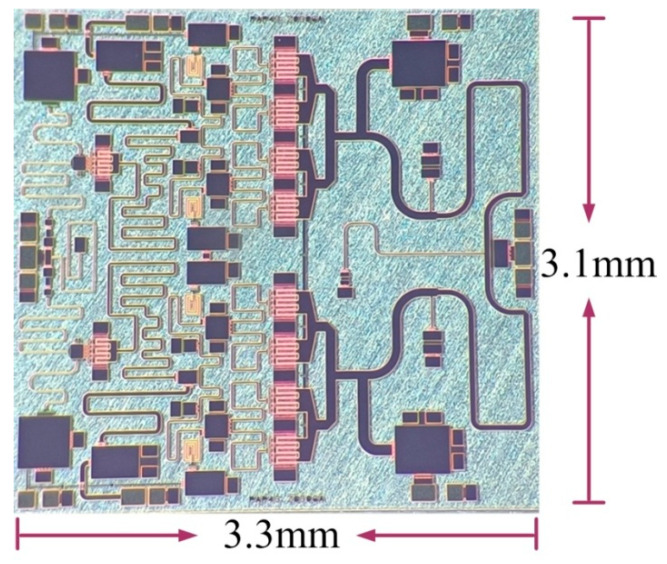
The photograph of the proposed 30 W PA MMIC.

The photograph of the proposed 30 W PA MMIC is shown in Figure 5. The horizontal dimension is 3.3 mm, and the vertical dimension is 3.1 mm. The PA MMIC is assembled into a fixture for measurement, and the back metal is pasted onto the aluminum shell through the nano silver conductive adhesive material. The proposed 30 W PA MMIC is characterized by small-signal and large-signal measurements to evaluate its performance at room temperature. The measurement is conducted under the condition of 100 µs pulse width and 10% duty cycle. The drain bias voltage is 28 V, and the gate bias voltage is −2.2 V.

**Figure 6:**
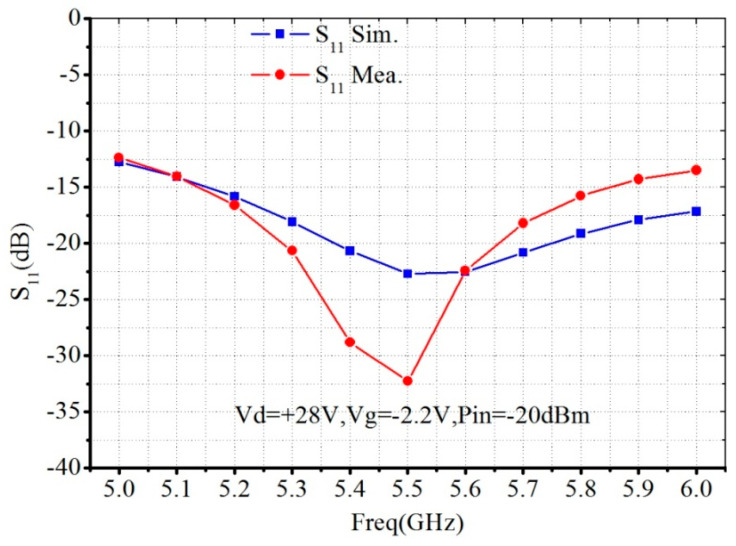
Simulated and measured *S*_11_ of the proposed C-band 30 W PA MMIC.

**Figure 7:**
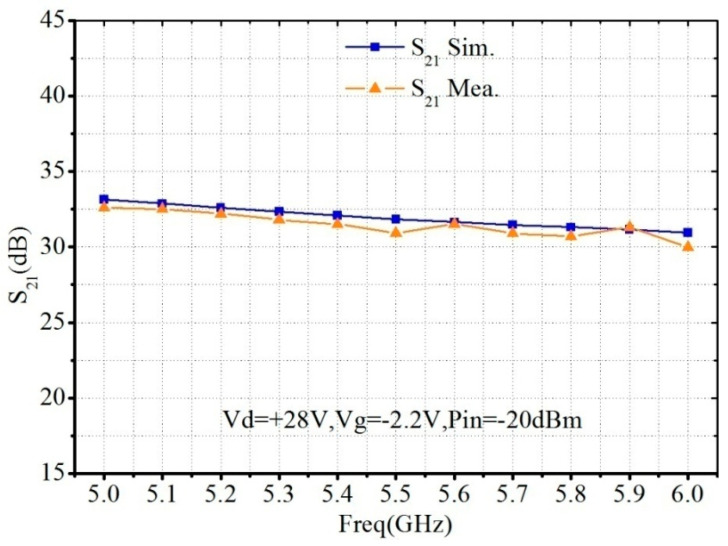
Simulated and measured *S*_21_ of the proposed C-band 30 W PA MMIC.

Figure 6 and Figure 7 show the small signal characteristic simulation and measurement results. The input return loss is less than −12 dB, the linear gain is about 32 dB, and the gain flatness is 2.5 dB.

**Figure 8:**
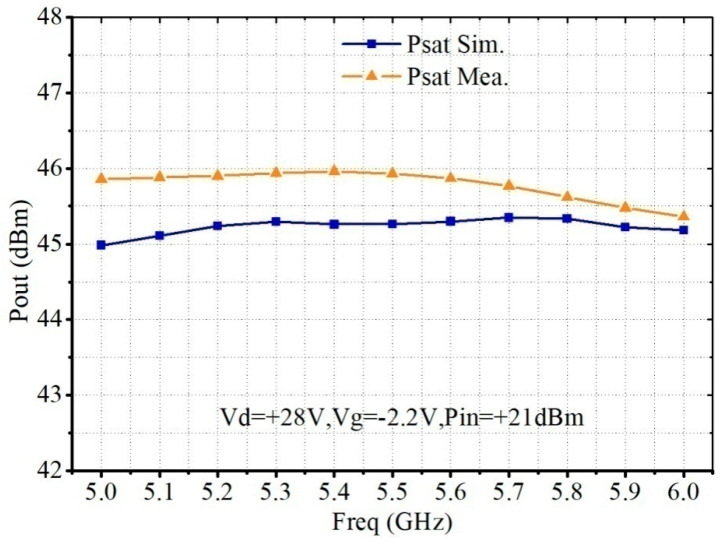
Simulated and measured saturated output power of the proposed C-band 30 W PA MMIC.

Figure 8 shows the saturated output power simulation and measurement results. As the input power of the PA MMIC is 21 dBm, the output power is greater than 45 dBm with 0.6 dB output power flatness.

**Figure 9:**
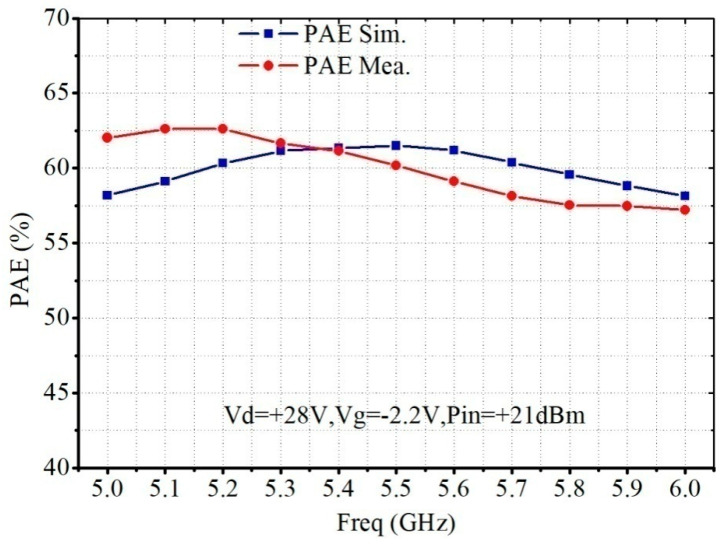
Simulated and measured PAE of the proposed C-band 30 W PA MMIC.

Figure 9 shows that the PAE simulation and measurement results. The PAE is more than 60% in the 5.0–5.6 GHz frequency range and more than 57% in the 5.0–6.0 GHz frequency range.

**Figure 10:**
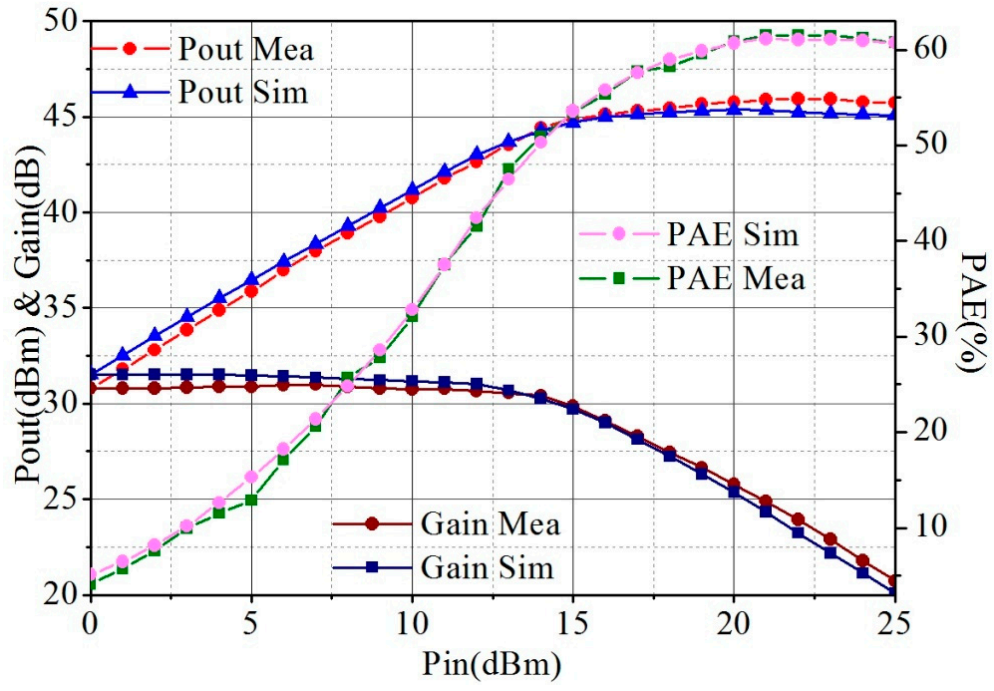
Simulated and measured Pout, PAE, and Gain curves versus Pin with 100 µs pulse width and 10% duty cycle at 5.5 GHz.

Figure 10 shows the comparison results of simulated and measured output power (Pout), PAE, and Gain curves versus input power (Pin). The test conditions were routine with 100 µs pulse width and 10% duty cycle at 5.5 GHz. The PAE exceeded 50% at the Pin fallback 6 dB.

**Figure 11:**
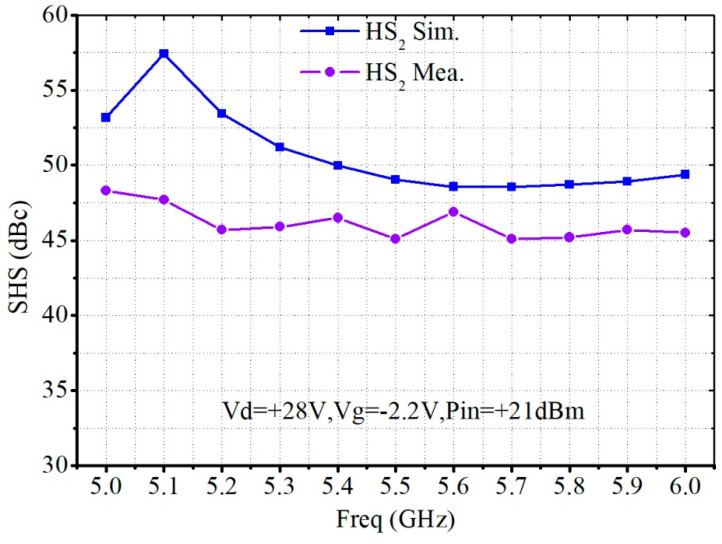
Simulated and measured SHS of the proposed C-band 30 W PA MMIC.

Figure 11 shows the SHS simulation and measurement results. The measured SHS was more than 45 dBc in the 5.0–5.6 GHz frequency range. However, compared with the simulation, the SHS measured deteriorated by more than 5 dB.

**Figure 12:**
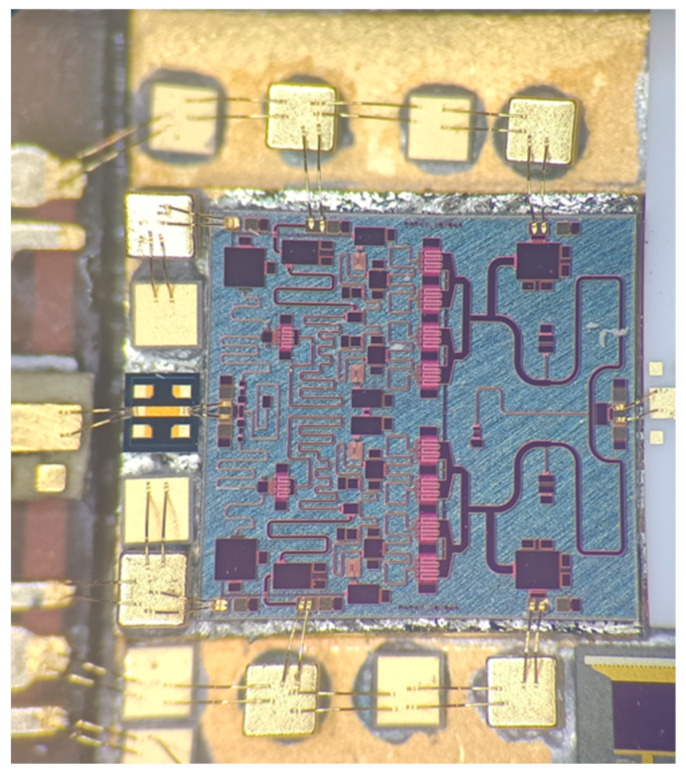
The partially enlarged picture of the proposed 30 W PA MMIC in the C-band module.

The PA MMIC was soldered into the C-band transceiver module, and the application environment of the C-band 30 W PA MMIC in the transceiver module was enlarged as shown in Figure 12. The gate and drain power supply pads are respectively bonded to the capacitors for filtering noise waves. The size of input and output RF signal pads is 150 × 100 µm, which was conducive to automatic double gold wire bonding. In the C-band module, the final measured results show that the output power of the C-band channel was more than 44.3 dBm, and the emission drain efficiency was more than 45%. Considering the total loss of about 0.8 dB caused by the circulator, microwave transmission line, SMA microwave connector, and the load impedance mismatch effect, it was consistent with the performance of the proposed PA MMIC.

Table 1 summarizes the performance comparison between the proposed 30 W PA MMIC and the state-of-the-art PA MMIC reported recently. Through the comparison of operating frequency bandwidth, output power, PAE, power gain, chip size, and SHS, it was found that the PA MMIC proposed in this paper has excellent comprehensive performance.

## 4. Discussion and Conclusions

In this paper, a high-performance C-band 30 W PA MMIC was designed based on 0.25 µm GaN HEMT technology. A method of combining high PAE with SHS was used. In order to optimize the PAE and SHS of the PA MMIC, the transistors and output matching network were combined with an integrated simulation design to improve the overall performance. The experimental results are in good agreement with the design simulation results, which verifies the feasibility of the proposed high PAE and SHS design method. In the 5–6 GHz frequency range, the power gain of the proposed PA MMIC is 24 dB, the gain flatness is less than ± 0.3 dB, the saturated output power is more than 45 dBm, the PAE is 57–61%, and the SHS is greater than 45 dBc. In addition, the overall size of the proposed C-band 30 W PA MMIC is only 3.3 × 3.1 mm^2^, which realizes excellent performance and meets the requirements proposed by the radar network system.

## Figures and Tables

**Table 1:** The performance comparison with other C-band PA MMICs in previous studies.

Reference	Frequency (GHz)	P_out_ (W)	PAE (%)	Power Gain (dB)	Die Area (mm^2^)	SHS (dBc)
[10]	5.5–6.2	50	35–42	22	3.8 × 3.9	--
[20]	5.6–6.3	30	59–62	21	4.2 × 4.0	--
[22]	5.0–5.8	40	41–45	21	4.5 × 4.0	--
[26]	5.0–6.0	50	42–46	20	4.3 × 4.3 *	>34
[27]	4.8–6.0	30	54–58	20	6.0 × 6.0 *	--
[28]	5.2–5.9	40	53–56	25	5.0 × 5.0 *	>29
[36]	5.0–6.0	60	42–45	25	3.2 × 5.3	--
This work	5.0–6.0	30	57–62	24	3.3 × 3.1	>45

* Package size.

## Data Availability

Not applicable.
